# Surgical Strategies for Unexpected Unilateral Absence of the Latissimus Dorsi Muscle during Breast Reconstruction: A Case Report and Literature Review

**DOI:** 10.1055/a-2820-5151

**Published:** 2026-05-29

**Authors:** Matteo Meroni, Tania Panettella, Elena Frey, Elmar Fritsche

**Affiliations:** 1 Department of Plastic and Hand Surgery, Luzerner Kantonsspital, Lucerne, Switzerland

**Keywords:** breast reconstruction, free flap, latissimus, serratus, microsurgery

## Abstract

The latissimus dorsi (LD) muscle is considered one of the most reliable donor sites for autologous breast reconstruction, particularly in patients with prior oncologic treatment or comorbidities precluding microsurgical options. While its consistent anatomy typically permits straightforward surgical planning, rare anatomical anomalies can dramatically alter intraoperative decision-making. We report the case of a 69-year-old woman with a long oncologic history and multiple comorbidities who underwent delayed breast reconstruction using a pedicled LD flap, 1 year after radical mastectomy and axillary lymphadenectomy. Intraoperatively, the LD muscle was found to be completely absent on the left side, with no identifiable muscle belly and tendon. Immediate adaptation was required, and a serratus anterior muscle flap based on a thoracodorsal branch was used instead. Interestingly, retrospective review of a CT scan performed 2 years earlier for a pulmonary complaint revealed clear evidence of unilateral LD muscle absence, an oversight that underscores the importance of targeted imaging review. This case is one of only four reported in the literature, where unilateral absence of the LD muscle in a non-syndromic patient was encountered, and the only one intraoperative discovery during breast reconstruction. This highlights the critical need for anatomical vigilance and intraoperative adaptability in reconstructive surgery. When standard options fail, familiarity with alternative flaps and sequential planning are essential to achieving functional and aesthetic outcomes.

## Introduction

The latissimus dorsi (LD) muscle is a common donor in autologous breast reconstruction for its broad surface, robust thoracodorsal pedicle, and reliable anatomy, particularly in patients with prior oncologic treatment or higher risk of reconstructive failure. 1 The pedicled LD flap offers soft tissue coverage with acceptable volume and contour, and is often chosen when microsurgical options are contraindicated. 2

Complete unilateral absence of the LD is exceptionally rare. While muscular agenesis is more often reported for the pectoral muscles (e.g., Poland syndrome), true unilateral LD agenesis is exceedingly rare; only three cases have been described so far. 3

We present a unique and instructive case of a 69-year-old woman with a history of left breast carcinoma who was planned to receive an autologous reconstruction with a pedicled LD muscle flap. Intraoperatively, the LD was completely absent on the left side, necessitating a salvage procedure. A thoracic CT performed 2 years earlier for a pulmonary complaint, reviewed retrospectively, already showed the absence of the LD, a detail unnoticed at the time.

## Case

A 69-year-old woman presented for left breast reconstruction. Twenty-four years earlier, she underwent segmentectomy for DCIS (pTis pN0 M0) with adjuvant radiotherapy. She later developed an invasive carcinoma in the upper lateral quadrant of the same breast with suspicious axillary nodes on CT. Following multidisciplinary discussion, she underwent a radical mastectomy with axillary lymphadenectomy and adjuvant chemotherapy.

**Fig. 1:**
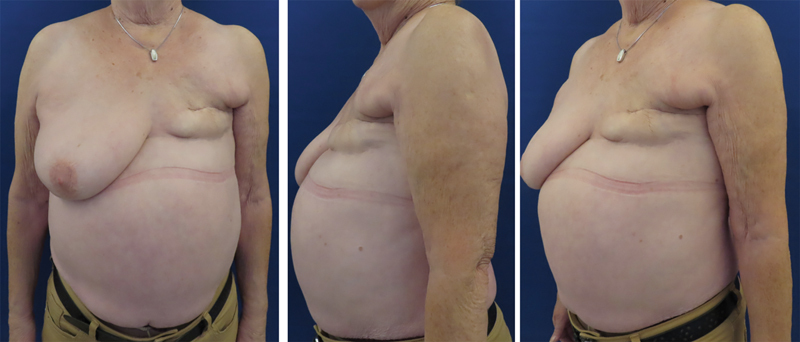
Preoperative pictures before the reconstructive procedure.

Early healing was complicated by wound dehiscence (managed conservatively) and a persistent seroma requiring aspirations. The chest wall healed flat with scar contracture, accentuating asymmetry with a large ptotic contralateral breast. Comorbidities (grade I obesity, heavy smoking history, daily alcohol intake) made a pedicled LD flap the preferred option. During the preoperative discussion, the patient explicitly refused a combined reconstruction with an implant. Microsurgical free flap reconstruction was deemed unsuitable. Symmetrization by contralateral reduction was planned. Surgery was scheduled 1 year after the mastectomy ( Fig. 1 ).

As the LD flap anatomy is highly consistent, no dedicated preoperative investigation was required. A standard skin paddle was designed, and dissection began uneventfully. Using the Olivari technique, 4 where the skin paddle is oriented almost perpendicular to the LD axis with circumferential initial incision, exposure of the anterior border proved limited. A serratus slip was misinterpreted as the LD, committing the team to a flawed dissection.

The plan was changed intraoperatively: A substantial portion of the serratus anterior was harvested. Axillary vascular dissection was challenging after a previous lymphadenectomy; the thoracodorsal artery and nerve were not reliably identified. The lateral thoracic artery and a serratus branch were dissected, and the flap was transferred. Although the volume was less than anticipated, perfusion was satisfactory on indocyanine green angiography.

**Fig. 2:**
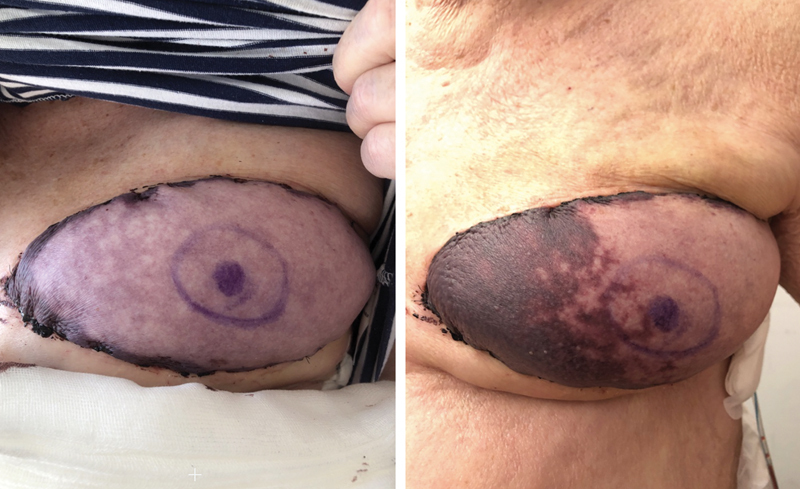
Clinical postoperative evolution of the flap.

**Fig. 3:**
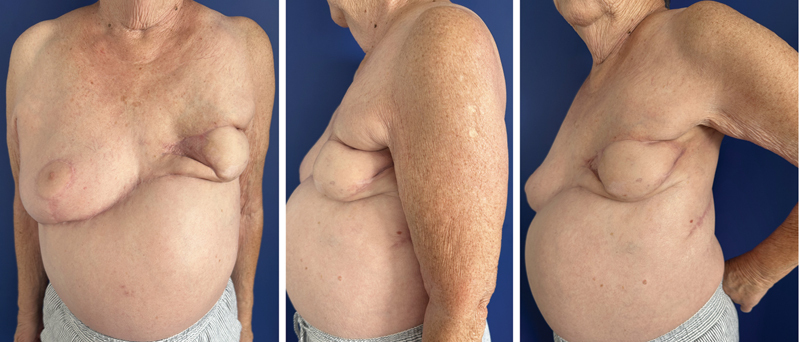
Postoperative pictures at 6 months follow-up.

Contralateral reduction was postponed. In the early postoperative period, venous congestion progressed to distal necrosis ( Fig. 2 ). The flap was monitored for 2 weeks. After demarcation, the necrotic segment was debrided, leaving a 6 × 4 cm defect. To close the defect and add volume, we attempted a propeller flap from the contralateral breast based on a random medial intercostal artery perforator. During the lower-pole resection of the contralateral breast, designed according to the planned breast reduction markings, we actually identified a suitable perforator in a distal–medial position. This would have enabled us to use the tissue that would otherwise have been discarded to try to reconstruct the affected breast. Despite adequate initial vascularity, tunneling and inset caused venous stasis; the flap was removed intraoperatively and the defect covered with a split-thickness skin graft. Healing was otherwise uneventful. At 6-month follow-up, the site was stable, and the patient was satisfied ( Fig. 3 ). Lipofilling was offered but declined.

**Fig. 4:**
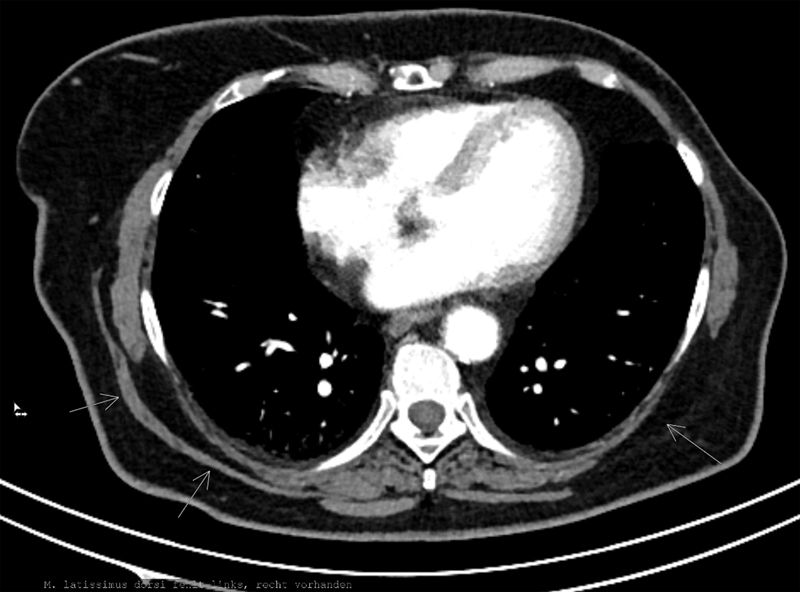
Preoperative (2 years before) CT scan, showing the unilateral latissimus dorsi muscle absence. CT, computed tomography.

Postoperatively, review of prior imaging revealed a thoracic CT performed 2 years earlier that clearly showed unilateral absence of the left LD muscle before any surgery or radiation in that region ( Fig. 4 ).

## Discussion

Intraoperative discovery of complete LD absence during a planned pedicled flap is profoundly uncommon. Our patient had no complaints of shoulder dysfunction and no suspicious thoracic contour, so a routine LD flap was expected. The lack of identifiable muscle fibers and tendons forced immediate revision of the reconstructive plan.

Erdem et al 5 reported a 73-year-old cadaver in whom the left LD was not identifiable during routine dissection, with only small residual fibers from lower thoracic segments and a thin, fragile fascia spanning approximately T6 to T12; no abnormalities were noted in the contralateral LD. In the same specimen, the thoracodorsal nerve was absent, and arterial branches from the subscapular/axillary system supplying the LD were not identified. This case would be coherent with the history of our patient.

Izadpanah et al 6 described an 82-year-old woman with prior left mastectomy, axillary lymph node dissection, and radiotherapy for breast carcinoma; years later, she underwent curative resection of a posterior shoulder/scapular border sarcoma, leaving a 15 × 20 cm defect with exposed scapula, for which a pedicled LD reconstruction was planned. Intraoperatively, the left LD was completely absent, and further dissection showed no tendon remnants or muscle fibers at origin or insertion; the surrounding musculature was symmetric. Retrospective review noted that the preoperative chest CT already demonstrated LD absence, although it had not been recognized preoperatively. Reconstruction was completed using a fasciocutaneous thoracodorsal artery perforator flap, with an uneventful postoperative course.

Seoyoun et al 7 reported a 73-year-old woman with a radiation-induced chronic right chest ulcer occurring 36 years after modified radical mastectomy and radiotherapy, with upper-extremity lymphedema for 30 years. Preoperative CT documented the absence of the right LD while confirming patency/presence of the right thoracodorsal artery, and an LD flap was initially planned. At surgery, no LD muscle was present beneath the designed skin paddle, and coverage was achieved with two sequential freestyle perforator flaps: An advanced intercostal artery perforator flap (using the original skin paddle territory) and a transposed thoracodorsal artery perforator flap.

Preoperative imaging review for flap-based reconstruction should include scrutiny of muscle volume and morphology when available. Even intact thoracodorsal vessels do not guarantee a functional LD. In patients with prior axillary surgery or radiation, dedicated radiological assessment of the LD should always be considered.

Among the possible differential diagnoses that could account for the unexpected unilateral absence of the LD muscle is prior iatrogenic injury to the thoracodorsal nerve. This possibility is particularly important to consider when planning secondary reconstruction after previous breast and axillary lymph node surgery, and radiotherapy may further contribute to muscular atrophy. However, this explanation does not apply in our case, as shown by the initial CT imaging.

The implications are substantial for comorbid patients in whom the LD flap may represent the final reliable option. In this setting, obesity, heavy smoking history, alcohol use, and a preference to avoid prolonged hospitalization, LD was the safest choice. Its unavailability required rapid pivoting to salvage alternatives. Harvesting a large serratus anterior flap based on a thoracodorsal branch 8 provided acceptable coverage; despite partial necrosis and subsequent debridement, stable reconstruction was achieved, and the patient declined further fat grafting.

This experience underscores the need for contingency planning and intraoperative flexibility. When the LD is unavailable, alternatives include serratus anterior, perforator-based, or parascapular flaps, and, when necessary, skin grafting. In irradiated or scarred fields, local tissue quality can limit options, so familiarity with multiple techniques and prompt decision-making are essential in high-risk patients.

Functionally, the patient reported no shoulder weakness or asymmetry, implying long-standing compensation. This mirrors earlier observations and invites study of the biomechanical impact of congenital LD absence. The potential association with spinal alignment, as in Erdem et al's cadaveric scoliosis, remains unclear. 5

In summary, this case emphasizes thorough preoperative imaging review, vigilance for rare anatomic variants, and readiness to shift to alternative reconstructive strategies. Serratus anterior and perforator flaps can provide satisfactory salvage when the LD is absent, avoiding more complex interventions and aligning with patient comorbidities and preferences.
